# Study on the effects of intestinal flora on gouty arthritis

**DOI:** 10.3389/fcimb.2024.1341953

**Published:** 2024-08-08

**Authors:** Niqin Xiao, Xiaoyu Zhang, Yujiang Xi, Zhenmin Li, Yuanyuan Wei, Jiayan Shen, Lin Wang, Dongdong Qin, Zhaohu Xie, Zhaofu Li

**Affiliations:** Yunnan University of Chinese Medicine, Kunming, China

**Keywords:** gouty arthritis, intestinal flora, pathogenesis, drug therapy, traditional Chinese medicine

## Abstract

Gouty arthritis (GA), a metabolic and immunologic disease, primarily affects joints. Dysbiosis of intestinal flora is an important cause of GA. The metabolic disorders of intestinal flora leading to GA and immune disorders might play an important role in patients with hyperuricemia and established GA. However, the exact mechanisms, through which the dysbiosis of intestinal flora causes the development of GA, are not fully understood yet. Moreover, several therapies commonly used to treat GA might alter the intestinal flora, suggesting that modulation of the intestinal flora might help prevent or treat GA. Therefore, a better understanding of the changes in the intestinal flora of GA patients might facilitate the discovery of new diagnostic and therapeutic approaches. The current review article discusses the effects of intestinal flora dysbiosis on the pathogenesis of GA and the cross-regulatory effects between gut flora and drugs for treating GA. This article also highlights the modulatory effects of gut flora by traditional Chinese medicine (TCM) to lower uric acid levels and relieve joint pain as well as provides a summary and outlook, which might help guide future research efforts.

## 1 Introduction

Studies have shown that the dysbiosis of intestinal flora induces gouty arthritis (GA) in people along with certain genetic and environmental factors (FitzGerald et al., 2020). GA, the most common inflammatory disease, is caused by abnormal purine metabolism, leading to metabolic and immunological imbalances (Jati et al., 2022). GA, an acute relapsing arthritis, is characterized by redness, swelling, and heat pain, often affecting single joints, such as the joints of the lower limbs. The initial symptoms appear in the first metatarsophalangeal joints. The epidemiological data of gout indicated a global prevalence of 1–4% with an incidence rate of 0.1–0.3%. Moreover, the prevalence rate is increasing yearly (Song et al., 2022). The male-to-female ratio ranges from 3:1–10:1, with a higher incidence rate in males (Ragab et al., 2017; Mbuyi and Hood, 2020). GA affects 3.9% of adults and 8.7% of people over 80 years of age, and current treatments may be ineffective, mainly due to the presence of comorbidities (Yang et al., 2024). The pathogenesis of GA is complex. It is currently believed that it arises from the combination of elevated levels of uric acid. This metabolic disease occurs when uric acid exceeds its saturation level in blood or tissue fluids, leading to the formation of monosodium urate (MSU) crystals. These crystals deposit locally in the joints, inducing inflammatory reactions and tissue destruction (Ragab et al., 2017; Mbuyi and Hood, 2020; Wang et al., 2024). However, the exact pathogenesis of GA remains unclear. Gut, the largest immune organ, contains over 100 trillion microbial cells, including more than 1,000 different species (De Sordi et al., 2017). It is known as the “second brain” or “second gene pool” (Ragab et al., 2017). Studies have suggested that numerous diseases are associated with disorders of the intestinal flora, which plays a crucial role in human metabolism and immune function (Cho and Blaser, 2012; Lynch and Pedersen, 2016; De Sordi et al., 2017; Ragab et al., 2017; Liu et al., 2023). Recent studies are emphasizing its distal effects and are not limited to the gut only (Consortium, H. M. P, 2012). Recent studies, both in animals and clinical settings, showed that changes in intestinal flora were associated with GA development, suggesting its use in monitoring the onset, progression, and recovery of GA (Milani et al., 2017; Surana and Kasper, 2017; Shin and Kim, 2018). This indicated the existence of a gut-joint inflammatory axis (Chen et al., 2023). It has been proposed that gut flora and its metabolites play an important role in several processes, including purine metabolism, extrarenal excretion of uric acid, protection of the intestinal barrier, and regulation of immune function (Shao et al., 2017; Bach Knudsen et al., 2018; Shin and Kim, 2018; Chu et al., 2021; Yin et al., 2022). The essential and conditionally essential amino acids, short-chain fatty acids (SCFAs), lipopolysaccharides (LPS), etc. are the main metabolites of intestinal flora. In the human body, intestinal flora metabolizes approximately 1/3 of uric acid. Intestinal flora secretes uric acid transporter protein, which transports uric acid from blood to the intestinal lumen. Moreover, physiological flora, including *Lactobacillus* produce SCFAs, thereby promoting the decomposition of uric acid; The intestinal flora of patients with GA has decreased physiological flora, such as *Bifidobacterium*, *Lactobacillus, butyric acid bacteria, Clostridium*, and *pre-cecal bacilli*. There is an increase in the opportunistic pathogenic flora, such as *Bacteroides、Bacteroides mucronosus* and *Bacteroides xylosus* (Siezen and Kleerebezem, 2011; Wu et al., 2011; Guo et al., 2016; Lin et al., 2020; Wen et al., 2020; Chu et al., 2021; Wu et al., 2021; Yang et al., 2021; Song et al., 2022). Briefly, GA patients exhibit changes in their intestinal flora, which are characterized by a decrease in physiologic flora and an increase in opportunistic pathogenic flora, leading to changes in intestinal flora metabolites.

The current study reviewed the effects of gut flora dysbiosis on GA pathogenesis and the cross-regulatory effects between gut flora and drugs used to treat GA. This study also discussed the modulatory effects on gut flora by traditional Chinese medicine (TCM) to lower uric acid and alleviate joint pain. It was hypothesized that intervening in the GA pathogenesis at an earlier stage through the gut-immunity-joint inflammation axis coupled with the development of novel strategies to treat GA might offer valuable insights for future research endeavors.

## 2 Influence of intestinal flora on the pathogenesis of GA

Several studies have demonstrated the role of intestinal flora in the pathogenesis of GA, which mainly includes purine metabolism disorders that affect uric acid levels, regulation of inflammatory factors and immune responses, and damage to the intestinal mucosal barrier.

### 2.1 Disorders in intestinal flora affect uric acid levels

The intestinal flora affects uric acid levels through several mechanisms. First, the excessive production of uric acid leads to an increase in essential and conditional amino acids in the metabolites of intestinal flora in GA patients, resulting in purine synthesis and metabolic disorders. At the same time, a large amount of xanthine oxidase (XOD) and LPS are produced. XOD oxidizes hypoxanthine and xanthine to uric acid, leading to the production of a large amount of uric acid (Liu et al., 2012; Vadakedath and Kandi, 2018). The GA patients exhibit higher levels of gram-negative bacteria, such as *Escherichia coli*. Moreover, LPS, a cell wall component of gram-negative bacteria, enhances the synthesis and activity of XOD (Shu and Mi, 2022). Second, disordered intestinal flora leads to a reduction in uric acid excretion. Studies have revealed that the principal transporters responsible for uric acid excretion are solute carrier family (SLC) 2 member 9 (SLC2A9) and ATP binding cassette subfamily G member 2 (ABCG2), and their expressions facilitate the excretion of uric acid (Merriman, 2015; Xu et al., 2016). However, in GA patients, impaired production of SCFAs by the intestinal flora results in reduced production of uric acid transporters and metabolites, such as hydrolases and uricase, by the intestinal epithelial cells. This decline subsequently lowers uric acid excretion (Merriman, 2015; Maiuolo et al., 2016; Xu et al., 2016; Pan et al., 2020; Yin et al., 2022). Therefore, the intestinal flora impacts purine metabolism, contributing to elevated uric acid levels, which in turn triggers the deposition of MSU crystals and initiates a cascade of immune-inflammatory reactions following deposition.

### 2.2 Inflammatory factors and regulation of the immune response

Researchers have examined the distal effects of intestinal flora and suggested that it can influence inflammation in GA patients by modulating the intestinal inflammatory response (Chang et al., 2014; Sun et al., 2017; Ratajczak et al., 2019; Balaguer et al., 2022; Chen et al., 2022; Gou et al., 2022). *Bifidobacterium* plays a key role in inhibiting the release of inflammatory factors (Xue et al., 2017; Li et al., 2022). Moreover, a reduction in the abundance of *Bifidobacterium* in the intestines of GA patients results in increased inflammatory factor release. Disrupted intestinal flora leads to abnormal activation of innate immune cells, thereby increasing the levels of the pro-inflammatory cytokines interleukin-12 (IL-12) and IL-23 and decreasing those of the anti-inflammatory cytokines, such as IL-10 and transforming growth factor β (TGF-β) (Zhang et al., 2020). SCFAs, the most common metabolites of intestinal flora, play a key role in immune regulation in GA. SCFAs facilitate the communication between the intestinal flora and immune system and can maintain the anti-inflammatory/pro-inflammatory balance (Ganapathy et al., 2013; Ratajczak et al., 2019). Moreover, SCFAs activate T lymphocytes and B lymphocytes, leading to the production of various inflammatory factors and antibodies. They also regulate the functions of intestinal macrophages and dendritic cells in immune response, primarily through the inhibition of inflammatory factors, promotion of regulatory T (Treg) cell differentiation, and mediation of reduced inflammation (Ratajczak et al., 2019). Additionally, SCFAs bind to and activate the nuclear transcription factor Peroxisome Proliferator-Activated Receptor γ (PPARγ), which antagonizes Nuclear Factor-κappa B (NF-κB) signaling, thereby inducing anti-inflammatory effects in the gut. *In vitro* studies demonstrated that SCFAs could reduce inflammation by inhibiting the activation of NF-κB and upregulating the expression levels of PPARγ in human HT-29 colonic epithelial cells (Bach Knudsen et al., 2018). In contrast, the GA patients with disrupted intestinal flora showed a reduced abundance of various probiotics, resulting in lower SCFA levels. This reduction led to a decrease in Treg cells (Milani et al., 2017; Zhou et al., 2018; Zheng et al., 2023) and a subsequent reduction in the expression levels of PPARγ (Zhou et al., 2018). LPS can activate the immune system via Toll-like receptors 4, which further activates macrophages and neutrophils, leading to increased production of tumor necrosis factor and interleukin1-β; this results in the activation of the inflammatory response (Loeser et al., 2022). LPS can also induce pyroptosis mediated by inflammasomes, such as Nod-like receptor pyrin domain containing 3 (Zhao et al., 2018), thus promoting the development of arthritis.

Distal effects of intestinal flora may play an important role in the immune mechanism of GA. Disturbed intestinal flora leads to a decrease in important beneficial bacteria such as bifidobacteria, a decrease in SCFAs, and an increase in LPS production, which leads to an anti-inflammatory/pro-inflammatory imbalance and the appearance of elevated levels of inflammatory factors, leading to the development and progression of GA.

### 2.3 Damage to the intestinal mucosal barrier

The intestinal mucosal system constitutes a significant portion of the human immune system and is closely related to the intestinal flora. The disrupted intestinal flora in GA patients damages the intestinal epithelial cells by producing toxic substances, including hydrogen sulfide, reactive oxygen species, reactive nitrogen species, etc., thereby reducing the protective barrier effect of the intestinal epithelial cells (Chen et al., 2022). The impaired function of the intestinal mucosal barrier triggers immune dysfunction, leading to the induction of more pro-inflammatory factors. *Bifidobacterium*, an intestinal probiotic, can improve the gastrointestinal mucosal barrier function by inhibiting harmful bacteria (Li et al., 2022). Moreover, SCFAs, the metabolites of intestinal flora, affect immune function by repairing the mucosal barrier (Zhou et al., 2018). In GA patients, both *Bifidobacteria* and SCFAs are reduced in the intestinal tract (Chu et al., 2021; Yang et al., 2021), while the disordered intestinal flora produces toxic substances. This leads to impaired mucosal barrier function, which cannot be restored in time, ultimately causing an increase in the pro-inflammatory factors.

## 3 Markers of intestinal flora for the diagnosis of GA

**Figure 1:**
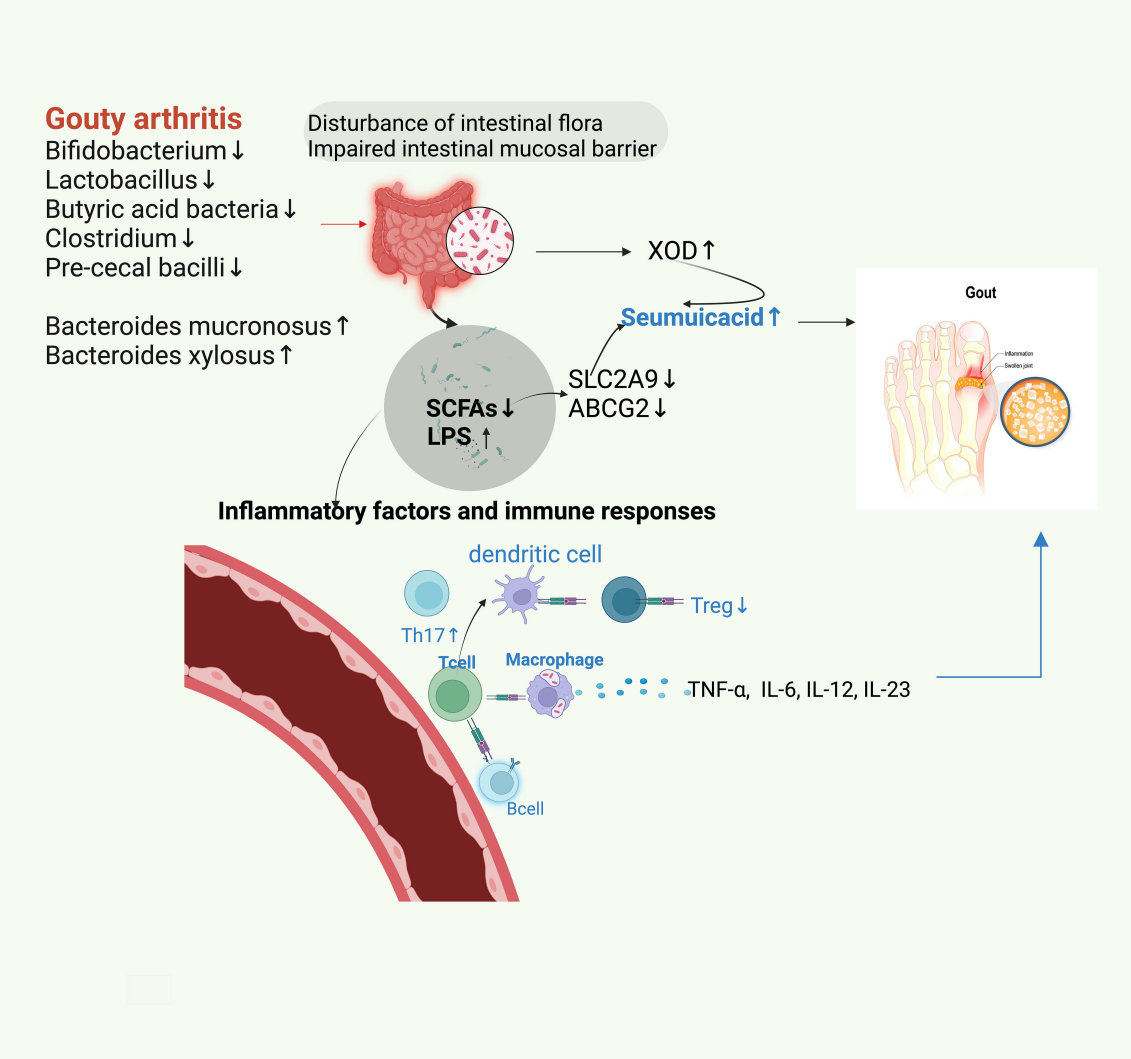
Dysbiosis of gut microbiota in patients with GA results in decreasing the abundance of physiologic microbiota and increasing that of opportunistic pathogenic microbiota. The disordered gut microbiota leads to impaired gastrointestinal mucosal barrier function, increased XOD and LPS, and decreased SCFAs. This reduces the contents of main uric acid transporters for uric acid excretion, SLC2A9, and ABCG2, thereby leading to an increase in uric acid and consequently triggering GA. The decrease in SCFA and the increase in LPS lead to the disorders of T-lymphocytes and B-lymphocytes, which cause an increase in IL-6, IL-12, and IL-13 levels. This disorder of the autoimmune system increases the proportion of TH17 cells and decreases that of Treg cells. Consequently, this phenomenon enhances inflammatory reactions and leads to GA.

Human gut is inhabited by more than 1,000 bacterial species; however, only 150–170 species are commonly found in the body (Patterson et al., 2016). Various diseases may be characterized by unique intestinal flora (Xu et al., 2020). Intestinal flora can be used as a non-invasive diagnostic and screening tool for diseases, including hepatocellular carcinoma and gastric cancer (Zha et al., 2023). Certain intestinal flora and their metabolites may be involved in GA by influencing uric acid metabolism, modulating inflammatory immune responses, and affecting the intestinal mucosal barrier, and therefore may be markers of intestinal flora for the diagnosis of GA. Studies have suggested that assessing the intestinal flora of GA patients might offer an earlier, more sensitive, and non-invasive method for detecting blood uric acid levels compared to conventional blood tests. Reduction in the *pre-cecal bacilli* and butyrate synthesis are the unique features of the gut flora in GA patients. A study by Zhuang Guo et al. identified an increase in the abundances of *Bacteroides mucronosus* and *Bacteroides xylosus* and a significant decrease in those of *E. faecalis* and *Bacteroides pseudoaceticus* in GA patients. Furthermore, a diagnostic model incorporating 17 GA-associated bacteria achieved an 88.9% accuracy rate in a validation group, consisting of 15 trial members, which was higher than that of the blood-uric acid-based method (Guo et al., 2016). Butyrate, one of the most common metabolites of intestinal flora, is a key communicator between intestinal flora and the immune system, playing a vital role in keeping the anti-inflammatory/pro-inflammatory balance. Moreover, it is one of the most extensively studied intestinal flora metabolites significantly associated with GA. Therefore, it is hypothesized that the reduction of *E. faecalis* anterior and the decrease in butyrate synthesis could serve as intestinal flora markers for diagnosing GA (**Figure 1**).

## 4 Cross-regulation between intestinal flora and drugs used to treat GA

### 4.1 Indirect regulation of intestinal flora

Studies suggested that the dynamics of the drug and intestinal flora could significantly affect the therapeutic effects of the drug (Džidić-Krivić et al., 2023). An increase in beneficial bacteria in the intestinal flora might serve as an indicator of therapeutic efficacy. Febuxostat is currently a commonly used uric acid-lowering drug, which could improve the limitation of intestinal flora biodiversity in GA patients (Wang et al., 2022). Rats treated with allopurinol and benzbromarone showed an increased abundance of Bifidobacteria in their intestinal flora (Maier et al., 2018; Yu et al., 2018). Maier L et al. showed that uric acid-lowering and anti-inflammatory drugs could partially restore intestinal flora after 24 weeks of treatment (Maier et al., 2018). Shi et al. (2020) observed that colchicine (COL) exposure induced a significant change in the diversity of the intestinal flora in mice (Jostins et al., 2012). Meanwhile, these disordered intestinal bacteria exhibited a significant dose-dependent effect. High doses of COL decreased the abundance of intestinal flora, indicating its antimicrobial potential. A significant shift in the dominant flora from the phylum *Bacteroidetes* to the phylum *Thick-walled* was observed under high-dose COL treatment, resulting in an increase in the ratio of *Thick-walled* to *Bacteroidetes*. Thick-walled phylum promotes the production of SCFAs and butyrate, which play a role in the treatment of GA by promoting uric acid excretion and inhibiting inflammatory factors (Jostins et al., 2012; Suzuki, 2013). Mäkivuokko et al. (2010) found that the long-term use of Nonsteroidal Antiinflammatory Drugs(NSAIDs) increased the abundance of the genus *Bacteroides* and decreased that of the Thick-walled phylum. Therefore, the short-term use of NSAIDs can restore the intestinal flora to normal, which may be one of its mechanisms of action (2013). However, the long-term use of NSAIDs can increase the abundance of genus *Bacteroides* in the intestinal flora, which in turn can increase gastrointestinal risks; therefore, oral acid-suppressing and stomach-protecting medications are needed for the protection of patients taking long-term NSAIDs orally (Zádori et al., 2023).

### 4.2 Direct regulation of intestinal flora

Prebiotics play a vital role in enhancing human intestinal health by inducing changes in bacterial composition and promoting the production of SCFAs, which result in immune stimulation, improved intestinal barrier function, and alteration of intestinal flora composition to treat GA (Gao et al., 2020; Kondratiuk et al., 2020). Guo et al. (2021) treated KO (*Uox* knockout) mice with inulin, a fermentable dietary fiber. The results showed reduced levels of uric acid, increased expression levels of ABCG2 in the intestine, decreased expression levels and activity of hepatic XOD, and enhanced production of SCFAs. The effects of probiotics in treating GA are similar to that of prebiotics. Probiotics, comprising of live microorganisms, primarily strains of *Lactobacillus* and *Bifidobacterium*, possessed anti-inflammatory and blood uric acid-lowering properties in experimental mice studies (Cleophas et al., 2017). Ni et al. (2021) showed that increasing the number of *Lactobacillus* strains in mice led to reduced serum uric acid levels, decreased XOD activity, increased SCFA production, decreased LPS concentrations, ameliorated hepatic inflammation, and mild renal injury. Wu et al. (2021) found that the *Lactobacillus fermentum* JL-3 strain could reduce uric acid levels and inflammatory response factors in mice.

Fecal microbiota transplantation (FMT) is a therapeutic approach that involves transferring physiological flora from the feces of healthy individuals into the gastrointestinal tract of patients to treat related diseases. It has emerged as a promising field of clinical investigation (Xie et al., 2022). Leichang Zhang et al. showed that FMT significantly decreased the helper T cells (Th) 1 and Th17 cells and reduced the levels of interferon-γ, IL-2, and IL-17. However, it significantly increased Th2 and regulatory T cells (Treg cells) as well as IL-4, IL-10, and TGF-β levels. Furthermore, the study observed an improvement in routine blood cell count in mice following FMT treatment (Zhang et al., 2021). Xie et al. (2022) conducted a study involving humans, where they explored wash-mass transplantation (WMT). Their research indicated that WMT resulted in reduced uric acid levels, a decreased frequency of joint pain episodes, shortened episode duration, and an improvement in intestinal barrier function (Xie et al., 2022). While the specific mechanisms and effects of FMT and WMT in the treatment of hyperuricemia and GA are unclear and still need further investigation, they offer valuable avenues for exploring new approaches to treat GA.

Dietary modification is a very important step in treating GA patients. Dietary fiber constitutes a significant component of their daily nutritional intake and consists of undigested food components in plant cell walls, including non-starch polysaccharides, lignin, etc (van der Beek et al., 2017). Dietary fibers serve as the substrate for anaerobic fermentation by intestinal flora and are mostly broken down into SCFAs, predominantly butyrate, propionate, etc (Ma et al., 2018). Butyrate acts as an agonist for certain G protein-coupled receptors, facilitating the conversion of naïve CD4+ T cells into immunosuppressive Tregs (Chen and Li, 2020). This process aids in inhibiting inflammatory factors, promoting Treg cell differentiation, and mediating inflammatory regression.

### 4.3 Research on Chinese medicine regulating intestinal flora to lower uric acid and improve arthritis

**Figure 2:**
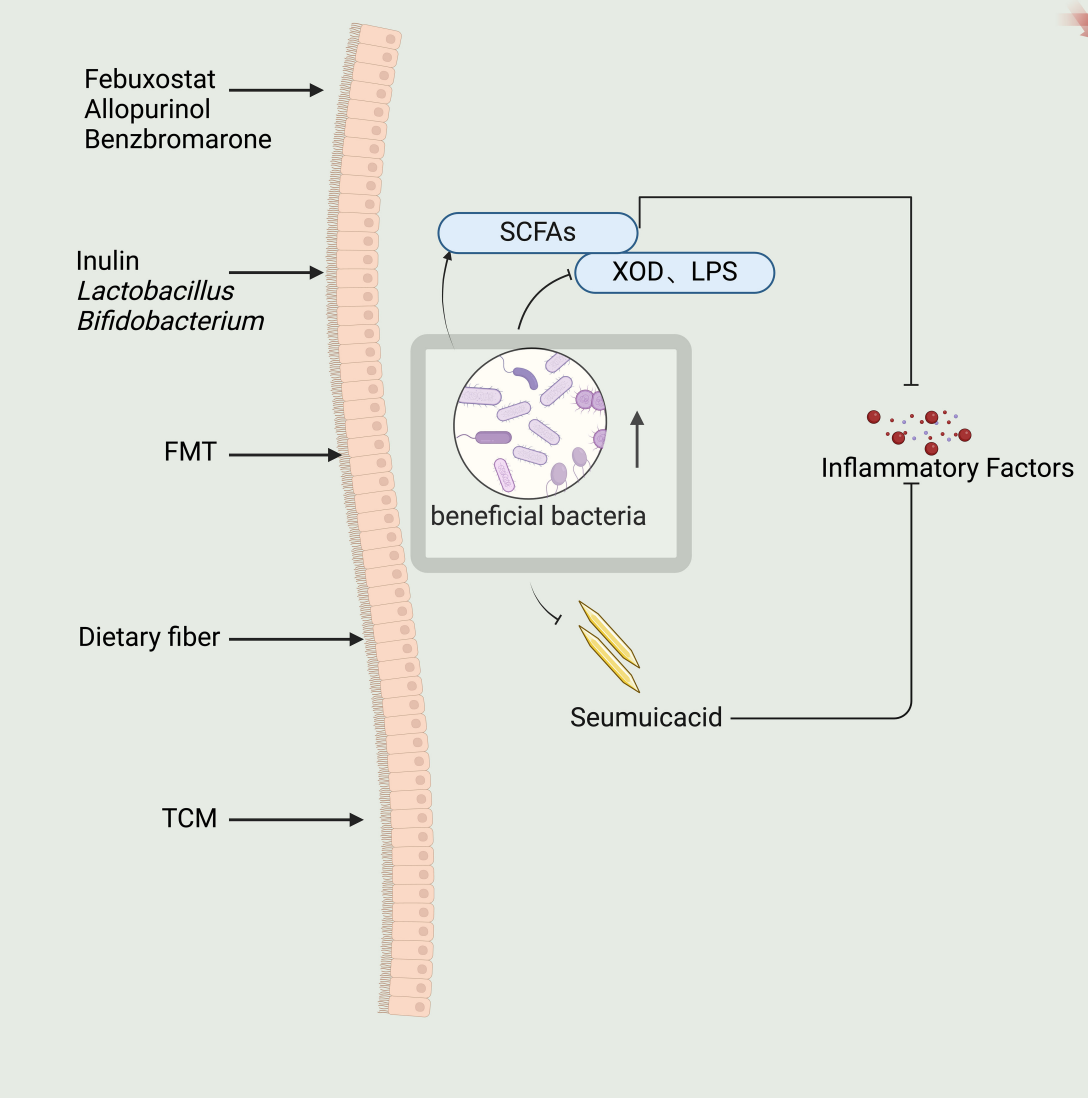
Indirect regulation of intestinal flora by febuxostat, allopurinol, benzbromarone and direct regulation of intestinal flora by inulin, Lactobacillus, Bifidobacterium, FMT and dietary fiber, as well as the effect of traditional Chinese medicine (TCM) on the intestinal flora can restore the beneficial intestinal flora and down-regulate the Seumuicacid, XOD, LPS, up-regulate the SCFAs, and ultimately down-regulate the secretion of inflammatory factors.

The potential mechanisms of several TCMs to treat GA have been linked to their ability to regulate the patient’s intestinal flora (Lin et al., 2020; Wang et al., 2022; Lin et al., 2023). Studies demonstrated that the *Quzhuo Tongbi* Formula could improve the structure and abundance of intestinal flora (Song et al., 2023). Chen et al. (2020) revealed that the *Dendrobium officinalis six* formula could regulate intestinal flora, thereby decreasing LPS production and reducing the levels of blood uric acid. Moreover, it improved the intestinal mucous membrane barrier and inhibited the production of NF-κB, reducing the release of inflammatory factors. Research has shown that *resveratrol* in *Polygonum cuspidatum* can increase the intestinal physiological microbiota, inhibit inflammatory factors (Zhou et al., 2024); *Plantago* can inhibit XOD activity to achieve a uric acid-lowering effect (Liu et al., 2022). Lin et al. (2023) used FMT to transfer the intestinal flora of GA mice treated with Si Miao Formula or Allopurinol to blank GA mice in order to investigate the therapeutic effects of FMT on GA. The results showed that compared to Allopurinol, Si Miao Formula showed a greater impact on the intestinal flora by restoring the abundance of the genus *Aspergillus* and *Helicobacter pylori*. Wang et al. (2022) found that flavored Baihu Formula could restore the abundance of families *Lactobacillaceae* and *Bifidobacteriaceae* to normal. Thus, restoring the intestinal flora of GA patients to normal plays a role in treating GA (**Figure 2**).

## 5 Summary and outlook

Numerous studies have demonstrated the role of intestinal flora in several diseases. In GA, intestinal flora participates in purine metabolism, the inflammatory factors and immune responses, and the intestinal mucosal barrier. Moreover, various ingredients of drugs used to treat GA could regulate the function of immune cells and reduce uric acid by normalizing the composition of intestinal flora. The range of methods for treating GA, by restoring and improving the intestinal flora, is increasing day by day. Moreover, TCM presents promising avenues for targeting and regulating intestinal flora in GA treatment. In the future, it might be possible to treat GA by specifically targeting the intestinal flora with TCM. It can be hypothesized that this approach might potentially enable earlier intervention in the GA pathogenesis through the gut-immune-joint inflammation axis and the development of more therapeutic strategies. Meanwhile, Intestinal flora maybe used as a non-invasive diagnostic and screening tool for GA. While some specific intestinal flora variations have been associated with GA, the limited research in this area and the substantial inter-individual differences in intestinal flora emphasize the need for further investigations to determine the diagnostic and therapeutic potential of intestinal flora and its metabolites in GA.

## Author contributions

NX: Writing – original draft. XZ: Writing – original draft. YX: Writing – original draft. ZmL: Writing – original draft. YW: Writing – original draft. JS: Writing – original draft. LW: Writing – original draft. DQ: Writing – review & editing. ZX: Writing – review & editing. ZfL: Writing – review & editing.

## References

[B1] Bach Knudsen K. E. Lærke H. N. Hedemann M. S. Nielsen T. S. Ingerslev A. K. Gundelund Nielsen D. S.. (2018). Impact of diet-modulated butyrate production on intestinal barrier function and inflammation. Nutrients 1010, 1499. doi: 10.3390/nu10101499 PMC6213552 30322146

[B2] Balaguer F. Enrique M. Llopis S. Barrena M. Navarro V. Álvarez B.. (2022). Lipoteichoic acid from bifidobacterium animalis subsp. Lactis bpl1: A novel postbiotic that reduces fat deposition via igf-1 pathway. Microbial Biotechnol. 153, 805–816. doi: 10.1111/1751-7915.13769 PMC8913875 33620143

[B3] Chang P. V. Hao L. Offermanns S. Medzhitov R. (2014). The microbial metabolite butyrate regulates intestinal macrophage function via histone deacetylase inhibition. Proc. Natl. Acad. Sci. U.S.A. 1116, 2247–2252. doi: 10.1073/pnas.1322269111 PMC3926023 24390544

[B4] Chen C. Li H. (2020). The inhibitory effect of gut microbiota and its metabolites on colorectal cancer. J. Microbiol. Biotechnol. 3011, 1607–1613. doi: 10.4014/jmb.2002.02032 PMC9728159 32522960

[B5] Chen M. Lin W. Li N. Wang Q. Zhu S. Zeng A.. (2022). Therapeutic approaches to colorectal cancer via strategies based on modulation of gut microbiota. Front. Microbiol. 13. doi: 10.3389/fmicb.2022.945533 PMC9389535 35992678

[B6] Chen X. Ge H. Z. Lei S. S. Jiang Z. T. Su J. He X.. (2020). Dendrobium officinalis six nostrum ameliorates urate under-excretion and protects renal dysfunction in lipid emulsion-induced hyperuricemic rats. BioMed. Pharmacother. 132, 110765. doi: 10.1016/j.biopha.2020.110765 33120237

[B7] Chen Z. J. Cai Z. W. Zhuang P. Z. Li F. Cui W. G. Li Z. C. (2023). Living probiotic biomaterials for osteoporosis therapy. Biomed. Technol. 1, 52–64. doi: 10.1016/j.bmt.2022.11.007

[B8] Cho I. Blaser M. J. (2012). The human microbiome: At the interface of health and disease. Nat. Rev. Genet. 134, 260–270. doi: 10.1038/nrg3182 PMC3418802 22411464

[B9] Chu Y. Sun S. Huang Y. Gao Q. Xie X. Wang P.. (2021). Metagenomic analysis revealed the potential role of gut microbiome in gout. NPJ Biofilms Microbiomes 71, 66. doi: 10.1038/s41522-021-00235-2 PMC8352958 34373464

[B10] Cleophas M. C. Crişan T. O. Joosten L. A. B. (2017). Factors modulating the inflammatory response in acute gouty arthritis. Curr. Opin. Rheumatol. 292, 163–170. doi: 10.1097/BOR.0000000000000366 27941389

[B11] Consortium, H. M. P (2012). Structure, function and diversity of the healthy human microbiome. Nature 4867402, 207–214. doi: 10.1038/nature11234 PMC3564958 22699609

[B12] De Sordi L. Khanna V. Debarbieux L. (2017). The gut microbiota facilitates drifts in the genetic diversity and infectivity of bacterial viruses. Cell Host Microbe 226, 801–808.e3. doi: 10.1016/j.chom.2017.10.010 29174401

[B13] Džidić-Krivić A. Kusturica J. Sher E. K. Selak N. Osmančević N. Karahmet Farhat E.. (2023). Effects of intestinal flora on pharmacokinetics and pharmacodynamics of drugs. Drug Metab. Rev. 551–2, 126–139. doi: 10.1080/03602532.2023.2186313 36916327

[B14] FitzGerald J. D. Dalbeth N. Mikuls T. Brignardello-Petersen R. Guyatt G. Abeles A. M.. (2020). 2020 american college of rheumatology guideline for the management of gout. Arthritis Rheumatol. (Hoboken N.J.) 726, 879–895. doi: 10.1002/art.41247 32390306

[B15] Ganapathy V. Thangaraju M. Prasad P. D. Martin P. M. Singh N. (2013). Transporters and receptors for short-chain fatty acids as the molecular link between colonic bacteria and the host. Curr. Opin. Pharmacol. 136, 869–874. doi: 10.1016/j.coph.2013.08.006 23978504

[B16] Gao J. Azad M. A. K. Han H. Wan D. Li T. (2020). Impact of prebiotics on enteric diseases and oxidative stress. Curr. Pharm. Design 2622, 2630–2641. doi: 10.2174/1381612826666200211121916 32066357

[B17] Gou H.-Z. Zhang Y.-L. Ren L.-F. Li Z.-J. Zhang L. (2022). How do intestinal probiotics restore the intestinal barrier? Front. Microbiol. 13. doi: 10.3389/fmicb.2022.929346 PMC9330398 35910620

[B18] Guo Y. Yu Y. Li H. Ding X. Li X. Jing X.. (2021). Inulin supplementation ameliorates hyperuricemia and modulates gut microbiota in uox-knockout mice. Eur. J. Nutr. 604, 2217–2230. doi: 10.1007/s00394-020-02414-x PMC8137640 33104864

[B19] Guo Z. Zhang J. Wang Z. Ang K. Y. Huang S. Hou Q.. (2016). Intestinal microbiota distinguish gout patients from healthy humans. Sci. Rep. 6, 20602. doi: 10.1038/srep20602 26852926 PMC4757479

[B20] Jati G. A. K. Assihhah N. Wati A. A. Salasia S. I. O. (2022). Immunosuppression by piperine as a regulator of the nlrp3 inflammasome through mapk/nf-κb in monosodium urate-induced rat gouty arthritis. Vet. World 152, 288–298. doi: 10.14202/vetworld.2022.288-298 PMC8980401 35400961

[B21] Jostins L. Ripke S. Weersma R. K. Duerr R. H. McGovern D. P. Hui K. Y.. (2012). Host-microbe interactions have shaped the genetic architecture of inflammatory bowel disease. Nature 4917422, 119–124. doi: 10.1038/nature11582 PMC3491803 23128233

[B22] Kondratiuk V. E. Tarasenko O. M. Karmazina O. M. Taranchuk V. V. (2020). Impact of the synbiotics and urate-lowering therapy on gut microbiota and cytokine profile in patients with chronic gouty arthritis. J. Med. Life 134, 490–498. doi: 10.25122/jml-2020-0065 PMC7803318 33456597

[B23] Li B. Ding M. Liu X. Zhao J. Ross R. P. Stanton C.. (2022). Bifidobacterium breve ccfm1078 alleviates collagen-induced arthritis in rats via modulating the gut microbiota and repairing the intestinal barrier damage. J. Agric. Food Chem. 7046, 14665–14678. doi: 10.1021/acs.jafc.2c04602 36377740

[B24] Lin X. Shao T. Huang L. Wen X. Wang M. Wen C.. (2020). Simiao decoction alleviates gouty arthritis by modulating proinflammatory cytokines and the gut ecosystem. Front. Pharmacol. 11, 955. doi: 10.3389/fphar.2020.00955 32670069 PMC7327538

[B25] Lin X. Wang M. He Z. Hao G. (2023). Gut microbiota mediated the therapeutic efficiency of Simiao decoction in the treatment of gout arthritis mice. BMC Complement. Med. Ther. 23, 206. doi: 10.1186/s12906-023-04042-4 37344836 PMC10286402

[B26] Liu H. Fan Y. M. Zhong J. Malkoch M. Cai Z. W. Wang Z. T. (2023). Advance in oral delivery of living material. Biomed. Technol. 3, 26–39. doi: 10.1016/j.bmt.2022.12.003

[B27] Liu Y. Yu P. Sun X. Di D. (2012). Metabolite target analysis of human urine combined with pattern recognition techniques for the study of symptomatic gout. Mol. Biosyst. 811, 2956–2963. doi: 10.1039/c2mb25227a 22932763

[B28] Liu Z. Q. Sun X. Liu Z. B. Zhang T. Zhang L. L. Wu C. J. (2022). Phytochemicals in traditional chinese medicine can treat gout by regulating intestinal flora through inactivating nlrp3 and inhibiting xod activity. J. Pharm. Pharmacol. 747, 919–929. doi: 10.1093/jpp/rgac024 35640306

[B29] Loeser R. F. Arbeeva L. Kelley K. Fodor A. A. Sun S. Ulici V.. (2022). Association of increased serum lipopolysaccharide, but not microbial dysbiosis, with obesity-related osteoarthritis. Arthritis Rheumatol. (Hoboken N.J.) 74, 227–236. doi: 10.1002/art.41955 PMC8795472 34423918

[B30] Lynch S. V. Pedersen O. (2016). The human intestinal microbiome in health and disease. N. Engl. J. Med. 37524, 2369–2379. doi: 10.1056/NEJMra1600266 27974040

[B31] Ma Y. Hu M. Zhou L. Ling S. Li Y. Kong B.. (2018). Dietary fiber intake and risks of proximal and distal colon cancers: A meta-analysis. Medicine 9736, e11678. doi: 10.1097/MD.0000000000011678 PMC6133424 30200062

[B32] Maier L. Pruteanu M. Kuhn M. Zeller G. Telzerow A. Anderson E. E.. (2018). Extensive impact of non-antibiotic drugs on human gut bacteria. Nature 5557698, 623–628. doi: 10.1038/nature25979 PMC6108420 29555994

[B33] Maiuolo J. Oppedisano F. Gratteri S. Muscoli C. Mollace V. (2016). Regulation of uric acid metabolism and excretion. Int. J. Cardiol. 213, 8–14. doi: 10.1016/j.ijcard.2015.08.109 26316329

[B34] Mäkivuokko H. Tiihonen K. Tynkkynen S. Paulin L. Rautonen N. (2010). The effect of age and non-steroidal anti-inflammatory drugs on human intestinal microbiota composition. Br. J. Nutr. 103, 227–234. doi: 10.1017/S0007114509991553 19703328

[B35] Mbuyi N. Hood C. (2020). An update on gout diagnosis and management for the primary care provider. Nurse Practitioner 4510, 16–25. doi: 10.1097/01.NPR.0000696896.83494.fe 32956194

[B36] Merriman T. R. (2015). An update on the genetic architecture of hyperuricemia and gout. Arthritis Res. Ther. 171, 98. doi: 10.1186/s13075-015-0609-2 PMC4392805 25889045

[B37] Milani C. Duranti S. Bottacini F. Casey E. Turroni F. Mahony J.. (2017). The first microbial colonizers of the human gut: Composition, activities, and health implications of the infant gut microbiota. Microbiol. Mol. Biol. Rev. 814, e00036-17. doi: 10.1128/MMBR.00036-17 PMC5706746 29118049

[B38] Ni C. Li X. Wang L. Li X. Zhao J. Zhang H.. (2021). Lactic acid bacteria strains relieve hyperuricaemia by suppressing xanthine oxidase activity via a short-chain fatty acid-dependent mechanism. Food Funct. 1215, 7054–7067. doi: 10.1039/D1FO00198A 34152353

[B39] Pan L. Han P. Ma S. Peng R. Wang C. Kong W.. (2020). Abnormal metabolism of gut microbiota reveals the possible molecular mechanism of nephropathy induced by hyperuricemia. Acta Pharm. Sinica. B 102, 249–261. doi: 10.1016/j.apsb.2019.10.007 PMC7016297 32082971

[B40] Patterson E. Ryan P. M. Cryan J. F. Dinan T. G. Ross R. P. Fitzgerald G. F.. (2016). Gut microbiota, obesity and diabetes. Postgraduate Med. J. 921087, 286–300. doi: 10.1136/postgradmedj-2015-133285 26912499

[B41] Ragab G. Elshahaly M. Bardin T. (2017). Gout: An old disease in new perspective - a review. J. Adv. Res. 85, 495–511. doi: 10.1016/j.jare.2017.04.008 PMC5512152 28748116

[B42] Ratajczak W. Rył A. Mizerski A. Walczakiewicz K. Sipak O. Laszczyńska M. (2019). Immunomodulatory potential of gut microbiome-derived short-chain fatty acids (scfas). Acta Biochim. Polonica 661, 1–12. doi: 10.18388/abp.2018_2648 30831575

[B43] Shao T. Shao L. Li H. Xie Z. He Z. Wen C. (2017). Combined signature of the fecal microbiome and metabolome in patients with gout. Front. Microbiol. 8. doi: 10.3389/fmicb.2017.00268 PMC5318445 28270806

[B44] Shi Y. Li J. Yang P. Niu Z. Wei L. Chen L.. (2020). Colchicine increases intestinal permeability, suppresses inflammatory responses, and alters gut microbiota in mice. Toxicol. Lett. 334, 66–77. doi: 10.1016/j.toxlet.2020.09.018 33002524

[B45] Shin W. Kim H. J. (2018). Intestinal barrier dysfunction orchestrates the onset of inflammatory host-microbiome cross-talk in a human gut inflammation-on-a-chip. Proc. Natl. Acad. Sci. U.S.A. 11545, E10539–E10547. doi: 10.1073/pnas.1810819115 PMC6233106 30348765

[B46] Shu S. Mi W. (2022). Regulatory mechanisms of lipopolysaccharide synthesis in escherichia coli. Nat. Commun. 131, 4576. doi: 10.1038/s41467-022-32277-1 PMC9356133 35931690

[B47] Siezen R. J. Kleerebezem M. (2011). The human gut microbiome: Are we our enterotypes? Microbial Biotechnol. 45, 550–553. doi: 10.1111/j.1751-7915.2011.00290.x PMC3819005 21848611

[B48] Song J. Jin C. Shan Z. Teng W. Li J. (2022). Prevalence and risk factors of hyperuricemia and gout: A cross-sectional survey from 31 provinces in mainland China. J. Trans. Internal Med. 102, 134–145. doi: 10.2478/jtim-2022-0031 PMC9328039 35959454

[B49] Song S. Fan M. Wen X. Shi X. Lou Y. He Z.. (2023). Integrated network pharmacology and gut microbiome analysis to reveal the mechanism of Qu-Zhuo-Tong-Bi decoction against hyperuricemia and gout. J. Ethnopharmacol. 316, 116736. doi: 10.1016/j.jep.2023.116736\ 37286117

[B50] Song S. Lou Y. Mao Y. Wen X. Fan M. He Z.. (2022). Alteration of gut microbiome and correlated amino acid metabolism contribute to hyperuricemia and th17-driven inflammation in uox-ko mice. Front. Immunol. 13. doi: 10.3389/fimmu.2022.804306 PMC8858814 35197978

[B51] Sun M. Wu W. Liu Z. Cong Y. (2017). Microbiota metabolite short chain fatty acids, gpcr, and inflammatory bowel diseases. J. Gastroenterol. 521, 1–8. doi: 10.1007/s00535-016-1242-9 PMC5215992 27448578

[B52] Surana N. K. Kasper D. L. (2017). Moving beyond microbiome-wide associations to causal microbe identification. Nature 5527684, 244–247. doi: 10.1038/nature25019 PMC5730484 29211710

[B53] Suzuki T. (2013). Regulation of intestinal epithelial permeability by tight junctions. Cell Mol. Life Sci. 704, 631–659. doi: 10.1007/s00018-012-1070-x PMC11113843 22782113

[B54] Vadakedath S. Kandi V. (2018). Probable potential role of urate transporter genes in the development of metabolic disorders. Cureus 103, e2382. doi: 10.7759/cureus.2382 PMC5973493 29850377

[B55] van der Beek C. M. Dejong C. H. C. Troost F. J. Masclee A. A. M. Lenaerts K. (2017). Role of short-chain fatty acids in colonic inflammation, carcinogenesis, and mucosal protection and healing. Nutr. Rev. 754, 286–305. doi: 10.1093/nutrit/nuw067 28402523

[B56] Wang X. Long H. Chen M. Zhou Z. Wu Q. Xu S.. (2022). Modified baihu decoction therapeutically remodels gut microbiota to inhibit acute gouty arthritis. Front. Physiol. 13. doi: 10.3389/fphys.2022.1023453 PMC9798006 36589463

[B57] Wang Y. Chen Y. Song Y. Chen H. Guo X. Ma L.. (2024). The impact of mHealth-based continuous care on disease knowledge, treatment compliance, and serum uric acid levels in Chinese patients with gout. Randomized controlled trial. JMIR Mhealth Uhealth. 12, e47012. doi: 10.2196/47012 38623741 PMC11034422

[B58] Wang Z. Li Y. Liao W. Huang J. Liu Y. Li Z.. (2022). Gut microbiota remodeling: A promising therapeutic strategy to confront hyperuricemia and gout. Front. Cell. Infect. Microbiol. 12. doi: 10.3389/fcimb.2022.935723 PMC9399429 36034697

[B59] Wen X. Lou Y. Song S. He Z. Chen J. Xie Z.. (2020). Qu-zhuo-tong-bi decoction alleviates gouty arthritis by regulating butyrate-producing bacteria in mice. Front. Pharmacol. 11. doi: 10.3389/fphar.2020.610556 PMC7884811 33603667

[B60] Wu G. D. Chen J. Hoffmann C. Bittinger K. Chen Y.-Y. Keilbaugh S. A.. (2011). Linking long-term dietary patterns with gut microbial enterotypes. Sci. (New York N.Y.) 3346052, 105–108. doi: 10.1126/science.1208344 PMC3368382 21885731

[B61] Wu Y. Ye Z. Feng P. Li R. Chen X. Tian X.. (2021). Limosilactobacillus fermentum jl-3 isolated from “jiangshui” ameliorates hyperuricemia by degrading uric acid. Gut Microbes 131. doi: 10.1080/19490976.2021.1897211 PMC8007157 33764849

[B62] Xie W.-R. Yang X.-Y. Deng Z.-H. Zheng Y.-M. Zhang R. Wu L.-H.. (2022). Effects of washed microbiota transplantation on serum uric acid levels, symptoms, and intestinal barrier function in patients with acute and recurrent gout: A pilot study. Digestive Dis. (Basel Switzerland) 405, 684–690. doi: 10.1159/000521273 34872097

[B63] Xie Y.-C. Jing X.-B. Chen X. Chen L.-Z. Zhang S.-H. Cai X.-B. (2022). Fecal microbiota transplantation treatment for type 1 diabetes mellitus with malnutrition: A case report. Ther. Adv. Chronic Dis. 13, 20406223221117449. doi: 10.1177/20406223221117449 36003287 PMC9393929

[B64] Xu F. Fu Y. Sun T.-Y. Jiang Z. Miao Z. Shuai M.. (2020). The interplay between host genetics and the gut microbiome reveals common and distinct microbiome features for complex human diseases. Microbiome 81, 145. doi: 10.1186/s40168-020-00923-9 PMC7545574 33032658

[B65] Xu X. Li C. Zhou P. Jiang T. (2016). Uric acid transporters hiding in the intestine. Pharm. Biol. 5412, 3151–3155. doi: 10.1080/13880209.2016.1195847 27563755

[B66] Xue L. He J. Gao N. Lu X. Li M. Wu X.. (2017). Probiotics may delay the progression of nonalcoholic fatty liver disease by restoring the gut microbiota structure and improving intestinal endotoxemia. Sci. Rep. 7, 45176. doi: 10.1038/srep45176 28349964 PMC5368635

[B67] Yang H.-T. Xiu W.-J. Liu J.-K. Yang Y. Hou X.-G. Zheng Y.-Y.. (2021). Gut microbiota characterization in patients with asymptomatic hyperuricemia: Probiotics increased. Bioengineered 121, 7263–7275. doi: 10.1080/21655979.2021.1976897 PMC8806635 34590550

[B68] Yang N. J. Sun C. Dong C. Huang Y. T. Zhu Y. J. Gu Z. F. (2024). Emerging microfluidics for the modeling and treatment of arthritis. Eng. Regen. 5, 153–169. doi: 10.1016/j.engreg.2024.02.002

[B69] Yin H. Liu N. Chen J. (2022). The role of the intestine in the development of hyperuricemia. Front. Immunol. 13. doi: 10.3389/fimmu.2022.845684 PMC8907525 35281005

[B70] Yu Y. Liu Q. Li H. Wen C. He Z. (2018). Alterations of the gut microbiome associated with the treatment of hyperuricaemia in male rats. Front. Microbiol. 9. doi: 10.3389/fmicb.2018.02233 PMC6156441 30283432

[B71] Zádori Z. S. Király K. Al-Khrasani M. Gyires K. (2023). Interactions between NSAIDs, opioids and the gut microbiota - Future perspectives in the management of inflammation and pain. Pharmacol. Ther. 241, 108327. doi: 10.1016/j.pharmthera.2022.108327 36473615

[B72] Zha C. Peng Z. Huang K. Tang K. Wang Q. Zhu L.. (2023). Potential role of gut microbiota in prostate cancer: Immunity, metabolites, pathways of action? Front. Oncol. 13. doi: 10.3389/fonc.2023.1196217 PMC10231684 37265797

[B73] Zhang L. Ma X. Liu P. Ge W. Hu L. Zuo Z.. (2021). Treatment and mechanism of fecal microbiota transplantation in mice with experimentally induced ulcerative colitis. Exp. Biol. Med. (Maywood N.J.) 24613, 1563–1575. doi: 10.1177/15353702211006044 PMC8283255 33926254

[B74] Zhang X. Chen B. D. Zhao L. D. Li H. (2020). The gut microbiota: Emerging evidence in autoimmune diseases. Trends Mol. Med. 269, 862–873. doi: 10.1016/j.molmed.2020.04.001 32402849

[B75] Zhao L. R. Xing R. L. Wang P. M. Zhang N. S. Yin S. J. Li X. C.. (2018). NLRP1 and NLRP3 inflammasomes mediate LPS/ATP-induced pyroptosis in knee osteoarthritis. Mol. Med. Rep. 17, 5463–5469. doi: 10.3892/mmr.2018.8520 29393464

[B76] Zheng H. T. Zhao Q. Y. Ding Y. Ma S. X. Chen W. X. Qiu J. L.. (2023). Investigation of the relationships among respiratory syncytial virus infection, t cell immune response and intestinal flora. Eur. Rev. For Med. Pharmacol. Sci. 276, 2671–2678. doi: 10.26355/eurrev_202303_31804 37013785

[B77] Zhou L. Zhang M. Wang Y. Dorfman R. G. Liu H. Yu T.. (2018). Faecalibacterium prausnitzii produces butyrate to maintain th17/treg balance and to ameliorate colorectal colitis by inhibiting histone deacetylase 1. Inflamm. Bowel Dis. 249, 1926–1940. doi: 10.1093/ibd/izy182 29796620

[B78] Zhou Y. Zeng Y. Wang R. Pang J. Wang X. Pan Z.. (2024). Resveratrol improves hyperuricemia and ameliorates renal injury by modulating the gut microbiota. Nutrients 16, 1086. doi: 10.3390/nu16071086 38613119 PMC11013445

