# Corrigendum: Study on the effects of intestinal flora on gouty arthritis

**DOI:** 10.3389/fcimb.2024.1483349

**Published:** 2024-09-20

**Authors:** Niqin Xiao, Xiaoyu Zhang, Yujiang Xi, Zhenmin Li, Yuanyuan Wei, Jiayan Shen, Lin Wang, Dongdong Qin, Zhaohu Xie, Zhaofu Li

**Affiliations:** Yunnan University of Chinese Medicine, Kunming, China

**Keywords:** gouty arthritis, intestinal flora, pathogenesis, drug therapy, traditional Chinese medicine

In the published article, there was an error in affiliation. Instead of “Yunnan University of Traditional Chinese Medicine”, it should be “Yunnan University of Chinese Medicine”.

The authors apologize for this error and state that this does not change the scientific conclusions of the article in any way. The original article has been updated.

